# The synergistic interaction of thermal stress coupled with overstocking strongly modulates the transcriptomic activity and immune capacity of rainbow trout (*Oncorhynchus mykiss*)

**DOI:** 10.1038/s41598-020-71852-8

**Published:** 2020-09-10

**Authors:** Alexander Rebl, Tomáš Korytář, Andreas Borchel, Ralf Bochert, Joanna Ewa Strzelczyk, Tom Goldammer, Marieke Verleih

**Affiliations:** 1 Institute of Genome Biology, Leibniz Institute for Farm Animal Biology (FBN), Wilhelm-Stahl-Allee 2, 18196 Dummerstorf, Germany; 2 Institute of Aquaculture and Protection of Waters, Faculty of Fisheries and Protection of Waters, University of South Bohemia, Husova tř. 458/102, 370 05 České Budějovice, Czech Republic; 3 Sea Lice Research Centre (SLRC), Department of Biology, University of Bergen, Thormøhlensgate 55, 5008 Bergen, Norway; 4 Institute of Fisheries, State Research Centre of Agriculture and Fisheries, Mecklenburg-Vorpommern (LFA-MV), Südstraße 8, 18375 Born, Germany; 5 Institute of Immunology, Federal Research Institute for Animal Health, Friedrich-Loeffler-Institute, 17493 Greifswald-Insel Riems, Germany; 6 Faculty of Agriculture and Environmental Sciences, University of Rostock, 18059 Rostock, Germany

**Keywords:** Immunology, Molecular biology

## Abstract

The objective of the present study is to identify and evaluate informative indicators for the welfare of rainbow trout exposed to (A) a water temperature of 27 °C and (B) a stocking density of 100 kg/m^3^ combined with a temperature of 27 °C. The spleen-somatic and condition index, haematocrit and the concentrations of haemoglobin, plasma cortisol and glucose revealed non-significant differences between the two stress groups and the reference group 8 days after the onset of the experiments. The transcript abundance of almost 1,500 genes was modulated at least twofold in in the spleen of rainbow trout exposed to a critical temperature alone or a critical temperature combined with crowding as compared to the reference fish. The number of differentially expressed genes was four times higher in trout that were simultaneously challenged with high temperature and crowding, compared to trout challenged with high temperature alone. Based on these sets of differentially expressed genes, we identified unique and common tissue- and stress type-specific pathways. Furthermore, our subsequent immunologic analyses revealed reduced bactericidal and inflammatory activity and a significantly altered blood-cell composition in challenged versus non-challenged rainbow trout. Altogether, our data demonstrate that heat and overstocking exert synergistic effects on the rainbow trout’s physiology, especially on the immune system.

## Introduction

Stressors in aquaculture cover a broad and diverse range of abiotic and biotic factors including inappropriate temperature, oxygen content, salinity, pH value, high doses of ultraviolet light, inorganic and organic substances, conspecific competition and predators. The co-occurrence of multiple stressors in aquaculture facilities^1–3^ has various physiologic consequences for the farmed fish ranging from moderate alterations at the molecular level to increased disease susceptibility, behavioural disorders or even mortalities^3–5^. It is well established that adverse environments involving multiple stressors provoke a status of immunosuppression^6–8^ related to severe consequences for aquaculture industry^9–12^. In essence, steroid hormones and neuroamines transfer the stress signals and impair a variety of immune functions^13–15^, including the reduced viability, proliferation and migration of immune-cell populations^16^, limited phagocytic activity and decreased production of pro-inflammatory mediators and antibodies^17–19^. These exogenously induced disturbances of the fishes’ homeostasis are reflected in various physiological alterations such as changed concentrations of plasma proteins or the altered expression of specific genes.

For decades, numerous scientific studies have been concerned with the recording and assessment of stress-induced species-specific responses to find objective criteria that allow for the evaluation of an anthropogenic husbandry environment and development of guidelines for managing aquaculture facilities. Most of those studies focussed on the identification of potential biomarker genes and signalling pathways related to one particular stressor. In this regard, *Oncorhynchus* spp., which includes both Pacific salmon and trout, is one of the most investigated fish genera^20^. Particularly, the rainbow trout is not only a model species for immunological and toxicological studies but has been exposed, for instance, to a variety of challenging temperatures. While the thermal preference of this genus is well below 20 °C^21^, the experiments included ice-cold temperatures^22^, moderately challenging temperatures of 18–23 °C^23–27^ and critical temperatures of 25–28  °C^28–30^. Concerning crowding, different stocking densities up to 120 kg/m^3^ were investigated over different periods^31–36^. Taken together, the resulting data sets document that individual challenge factors produce distinct and only slightly overlapping molecular signatures. Although multiple studies^37–41^ addressed the impact of simultaneously changing environmental conditions on the physiology of bony fish, only a few studies integrated a broader and complementing panel of phenotypic, plasma- and transcript-based parameters. As transcriptomic analyses detect the entirety of modulated gene expressions and affected signalling pathways, they help to understand whether concurrent stressors evoke additive, synergistic or even antagonistic effects at the molecular level.

The present study addresses the question whether the combination of critical temperature and overstocking evokes a stress response in rainbow trout. Our major goal was to identify novel potential biomarkers and induced functional pathways which provide sufficient information to identify improper husbandry conditions of rainbow trout. Using separate experimental approaches in our previous studies, we investigated the impact of adverse temperature^23,27^ and different stocking densities^36^ on rainbow trout. This current study followed a similar experimental design, but we exposed rainbow trout simultaneously to a critical water temperature and crowding. A water temperature of 27 °C was chosen as the primary challenge factor. In the summer months of recent years, this temperature was repeatedly measured in the German coastal regions along the Baltic Sea where rainbow trout are bred for commercial and research purposes (Suppl. Fig. 1). The increase in water temperature is likely a consequence of global warming. An increased stocking density of 100 kg/m^3^ was selected as the secondary challenge factor. This stocking density represents the upper limit for the conventional production of rainbow trout of respective age and weight as recommended by the Food and Agriculture Organisation of the United Nations^42^. Our previous investigations on the impact of crowding on rainbow trout revealed that a stocking density of 120 kg/m^3^ significantly modulated the transcriptome of stress-relevant organs^36^ in comparison with those of trout kept at an uncrowded density^33^ of 30 kg/m^3^, which corresponds approximately to the density recommended for the organic aquaculture of trout (Commission Regulation (EC) No 710/2009 of 5 August 2009, Appendix XIIIa).

To investigate how a challenge-induced response influences the immune system, we focused on the major immune organs: the spleen, head kidney and peripheral blood leukocytes. The spleen was selected for transcriptomic analysis as it is a major secondary lymphoid organ that represents the “meeting point” for pathogens and immune cells and is thus characterised by a generally remarkable magnitude of responses^43–45^. The head kidney plays a central role in the haematopoiesis of fish, also as a major reservoir of myeloid cells, which act as first responders upon pathogenic invasion^46–48^. The head kidney’s ability to eliminate pathogens and orchestrate inflammatory responses has previously been studied under various conditions (diets and exposure to stressors and pathogens) as a reference for the immunocompetence and was investigated here in vitro after stimulation with the model pathogen *Aeromonas salmonicida*. Furthermore, we studied the composition of major subpopulations of circulatory leukocytes, which reflect the physiological and pathological stress-induced changes.

## Results

### Characterisation of the response to heat and crowding based on condition indices and blood parameters

**Figure 1:**
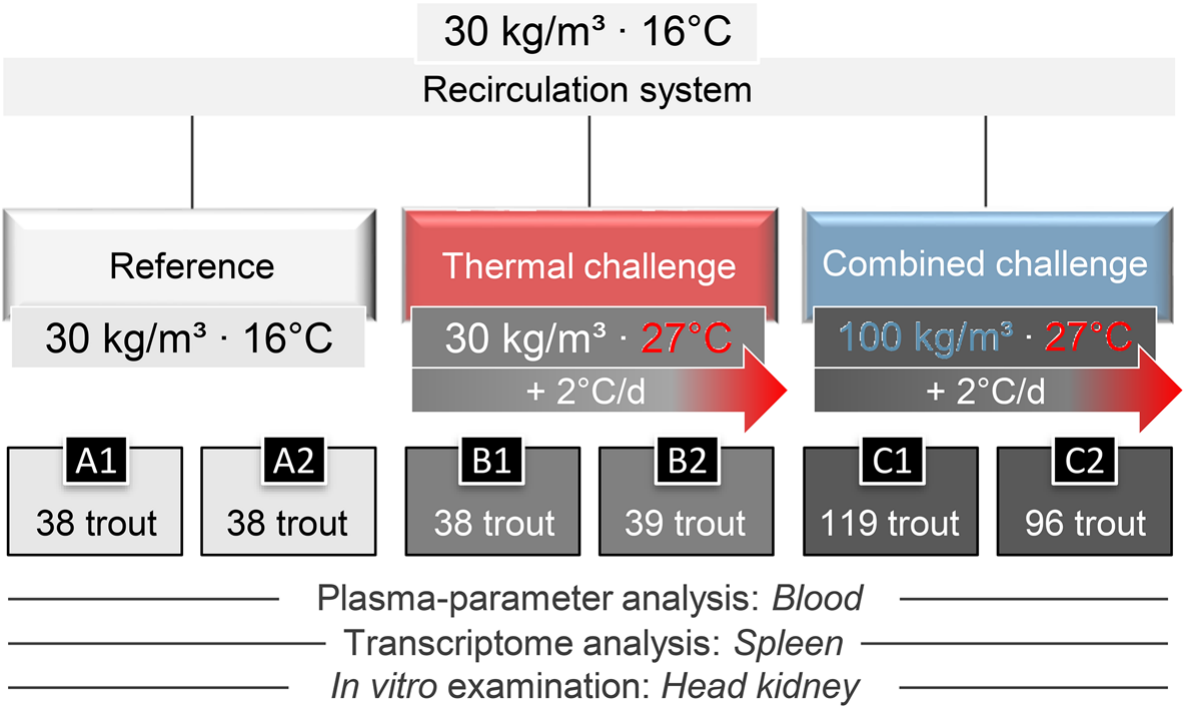
Schematic representation of the challenge experiment conducted on 368 rainbow trout. The total number of trout per experimental tank is indicated. In the reference tanks A1 and A2, the water temperature (16 °C) and the stocking density (30 kg/m^3^) were kept constant at the optimal range throughout the 8-day experiment. The thermal-challenge sub-experiment (labelled in red here and in the following figures) was performed in the tanks B1 and B2, where the water temperature was gradually increased to 27 °C while maintaining a stocking density of 30 kg/m^3^. The combined-challenge sub-experiment (labelled in blue) in tanks C1 and C2 exposed rainbow trout to a high stocking density of 100 kg/m^3^ while gradually increasing the water temperature to 27 °C. Blood samples, spleen and head kidney samples of seven trout from each tank were taken by the end of the experiment and used for the analysis as indicated below the scheme.

**Figure 2:**
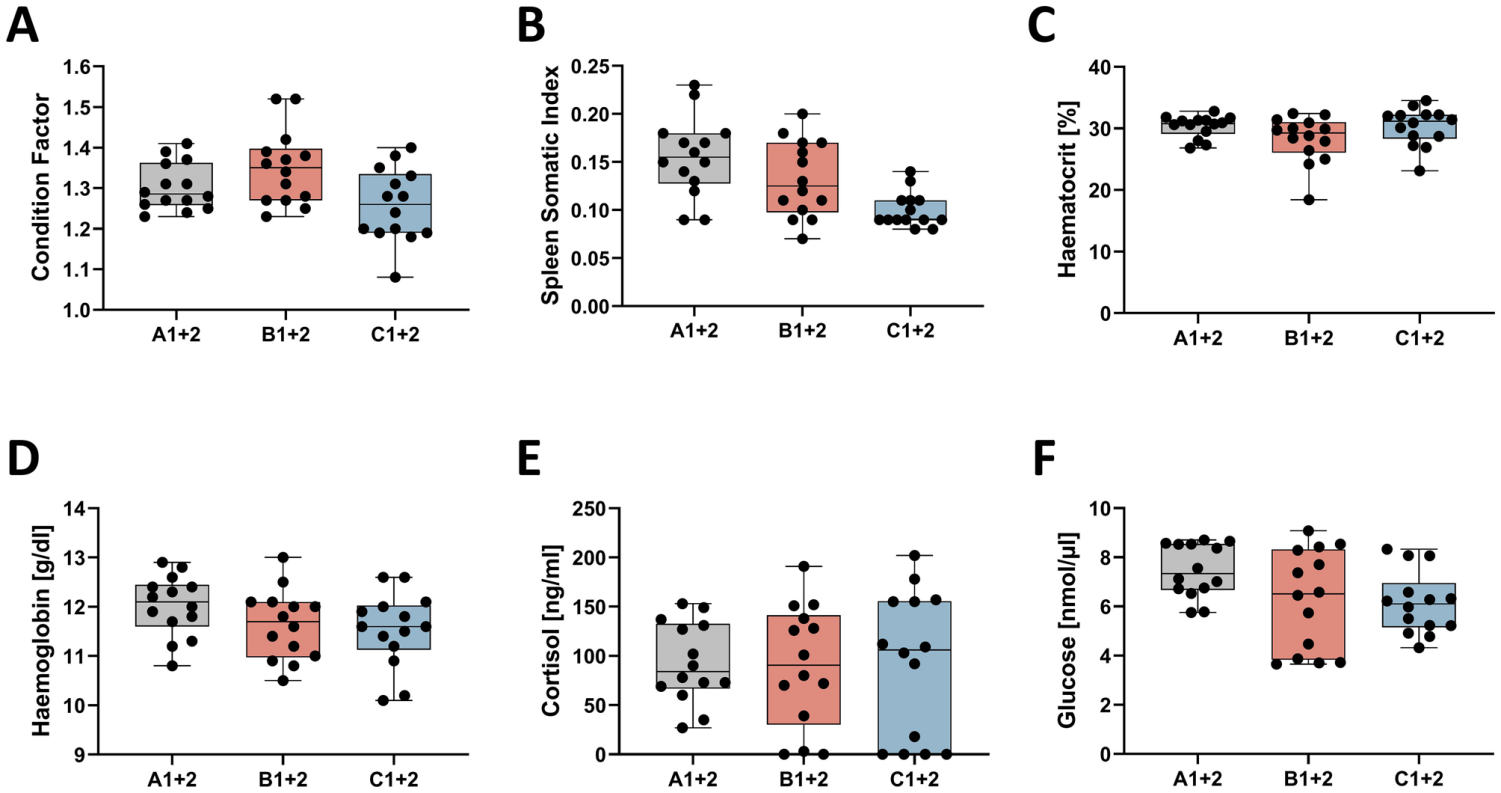
Condition indices and blood parameters. Box and whisker plots illustrating (**A**) condition factor, (**B**) spleen-somatic index, (**C**) haematocrit [%], (**D**) haemoglobin [g/dl], (**E**) cortisol concentration [ng/ml] and (**F**) glucose concentrations [nmol/µl] in plasma of 14 individuals from the experimental tanks as listed along the abscissa (*cf*. Figure 1). Individual measuring points are indicated by black dots; error bars indicate standard deviation.

Using a multi-parametric approach on rainbow trout cultivated in recirculating aquaculture system (RAS) tanks, we assessed the effects of a critical temperature (gradual temperature increase up to 27 °C; hereafter referred to as the thermal-challenge group B) compared to the combined effects of critical temperature and crowding (gradual temperature increase up to 27 °C at a stocking density of 100 kg/m^3^; hereafter referred to as combined-challenge group C) (Fig. 1). We determined the condition score, spleen-somatic index and haematocrit (Fig. 2A–C) and recorded the traditional and relatively fast detectable parameters of haemoglobin, cortisol and glucose concentration in the blood of rainbow trout (Fig. 2D–F) under challenging versus control conditions. Each of these tests revealed non-significant differences between the challenge groups B and C and the reference group A.

### Gene-expression profiling in the spleen of trout exposed to critical temperature

**Figure 3:**
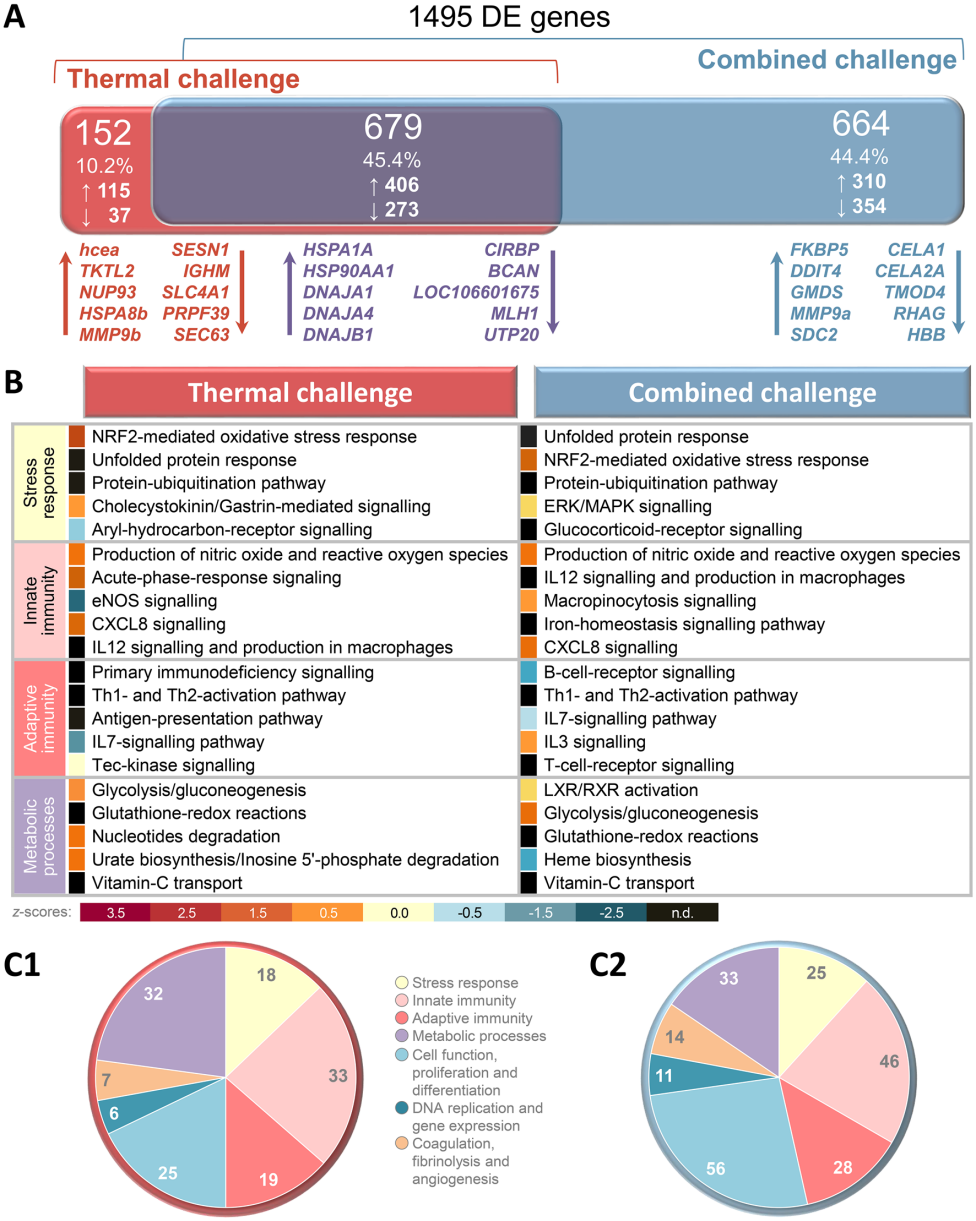
Classification of differently expressed genes. (**A**) Venn diagram illustrating the absolute number, the percentage and the number of up- (↑) and down-regulated (↓) DE features in the spleen of rainbow trout exposed to high temperature (red), or high temperature combined with crowding (blue) relative to the expression values obtained for the reference group. The overlap (purple) depicts the proportion of DE genes shared by both challenge groups. The diagram was calculated based on Agilent ID lists; the five most up- and downregulated genes in the respective lists are shown below the Venn diagram. (**B**) A pathway analysis of those genes differentially expressed after thermal challenge (left side of table) and after combined challenge (right side). The pathways were sorted according to the biofunction categories “stress response”, “innate immunity”, “adaptive immunity” and “metabolic processes”; the 5 most strongly affected pathways are listed. The colour code below the table indicates the z-score values. The total number of affected pathways was assigned to seven categories, which are shown in the two separate pie charts for (**C1**) temperature stress alone or (**C2**) combined temperature/crowding stress.

We detected a strong influence of both challenging conditions on the gene expression in the spleen. In total, the transcript abundance of almost 1,500 genes was modulated at least twofold for rainbow trout exposed to a high temperature alone or a high temperature combined with crowding as compared to the reference fish (Fig. 3A; Gene Expression Omnibus (GEO) accession code: GSE129271).

Gradual thermal rise to a temperature of 27 °C (thermal-challenge group B) caused the different regulation of 831 features in the spleen—i.e., 521 upregulated and 310 downregulated features (Fig. 3A)—relative to the reference group A, which was kept at 16 °C. Accordingly, approximately two-thirds (62.7%) of the entire set of differentially expressed (DE) genes in the spleen of rainbow trout exposed to high temperature was upregulated. These DE genes were assigned to 140 canonical pathways using the ingenuity pathway (IPA) programme. The three most highly temperature-affected pathways were assigned to the biofunction category “stress response”. These were (1) the *nuclear factor erythroid 2–related factor (2NRF2)-mediated oxidative stress response* (involving 27 DE genes), (2) *unfolded protein response* (14 DE genes) and (3) *protein ubiquitination pathway* (23 DE genes) (Fig. 3B).

Almost a quarter of the temperature-affected pathways (23.6%; *n* = 33) were attributed to the „innate immune system “ (Fig. 3C1). A similarly high proportion was assigned to the category „metabolic processes “ (22.9%; *n* = 32), whereas the number of pathways associated with the „adaptive immune system “ (13.6%; *n* = 19) and “stress response” (12.9%; *n* = 18) was significantly lower.

### Gene-expression profiling in the spleen of trout exposed to a high temperature combined with crowding

The combination of high temperature and crowding (combined-challenge group C) resulted in 1,343 DE genes in the spleen of challenged trout as compared to reference group A. In comparison with the expression data from the thermal-challenge group B versus group A, the upregulated genes increased in the combined-challenge group C by 37.4% to 716 DE genes, and the downregulated genes increased by 102.3% to 627 DE genes. Consequently, the ratio of up- and downregulated features after the combined challenge—accounting for 56.6% and 43.4%, respectively—revealed more balance than what was observed with high temperature alone.

The list of genes differentially expressed in response to the combined challenge was assigned to 213 canonical signalling pathways. As with the temperature-affected pathways, the *NRF2-mediated oxidative stress response* (involving 35 DE genes) and the *unfolded protein response* (19 DE genes) were the two major pathways affected by the combined challenge. More than a quarter of the pathways affected by high temperature and crowding (26.3%, *n* = 56) were attributed to the category “cell function, proliferation and differentiation” (Fig. 3C2). Approximately a fifth of all affected pathways were related to “innate immunity” (21.6%, *n* = 46). In comparison, the combined challenge modulated a significantly lower number of pathways associated with the “adaptive immune system” (13.1%; *n* = 28) and “stress response” (11.7%; *n* = 25).

### Identification of common and unique splenic pathways modulated by high temperature alone or by the combined temperature/crowding challenge

The two DE gene lists derived from the thermal- and combined-challenge sub-experiments share 679 common genes (Fig. 3A). Of these, the greater proportion of 406 genes (59.8%) was upregulated. Noteworthy, 10.1% of these upregulated features (*n* = 41) belong to the family of heat-shock protein (HSP)-encoding genes. These include the *HSP90/HSPC*, *HSP70/HSPA*, *HSP40/DNAJ* and *HSP47/SERPINH1* members. These HSP-encoding genes were also the most strongly upregulated features, while cold-inducible mRNA binding protein *(CIRBP)* was the most strongly downregulated gene (< -14-fold) in both gene sets (Fig. 3A). Moreover, 6.2% of the downregulated genes (*n* = 17) were associated with the heavy or light chain of the different immunoglobulin types in trout.

Accordingly, the analysis of the pathways induced by the thermal challenge and the combined challenge revealed that the typical “stress pathways” that are dependent on HSPs (the *unfolded protein response*, the *NRF2-mediated oxidative stress response* and the *protein-ubiquitination pathway*) and immunoglobulins (*primary immunodeficiency signalling*), were significantly affected by both experimental conditions (Fig. 3B). In addition to the biofunction cluster “stress response”, the categories “innate immunity” (*production of nitric oxide and reactive oxygen species*, *CXCL8 signalling*, *IL12-signalling and production in macrophages*), “adaptive immunity” (*Th1- and Th2-activation pathway*, *IL7-signalling pathway*) and “metabolic processes” (*glycolysis/gluconeogenesis*, *glutathione-redox reactions*, *vitamin-C transport*) contain pathways which were highly affected both by high temperature alone and by the combined challenge (Fig. 3B). In essence, the high proportion of commonly regulated genes is well reflected by a high degree of shared pathways, which indicates the various physiological aspects of a response to adverse water temperatures (Fig. 3C1) and the combination of adverse temperatures and stocking densities (Fig. 3C2). Nevertheless, it should be noted that the number of pathways belonging to the category „cell function, proliferation and differentiation “ has more than doubled in the combined-challenge dataset (*n* = 56, Fig. 3C2) compared with the thermal-challenge dataset (*n* = 25, Fig. 3C1).

The comparison of both DE-gene lists also revealed clusters of DE genes which were uniquely regulated either after thermal or after combined challenge (Fig. 3A). Critical temperature alone regulated 152 unique features (i.e., those genes not included in the DE gene list of the combined-challenge group), 115 (75.7%) of which were upregulated. In comparison, the combined challenge altered the expression of more than a fourfold number of unique genes (*n* = 664) with about a similar amount of up regulated features (*n* = 310) was there were downregulated features (*n* = 354).

**Figure 4:**
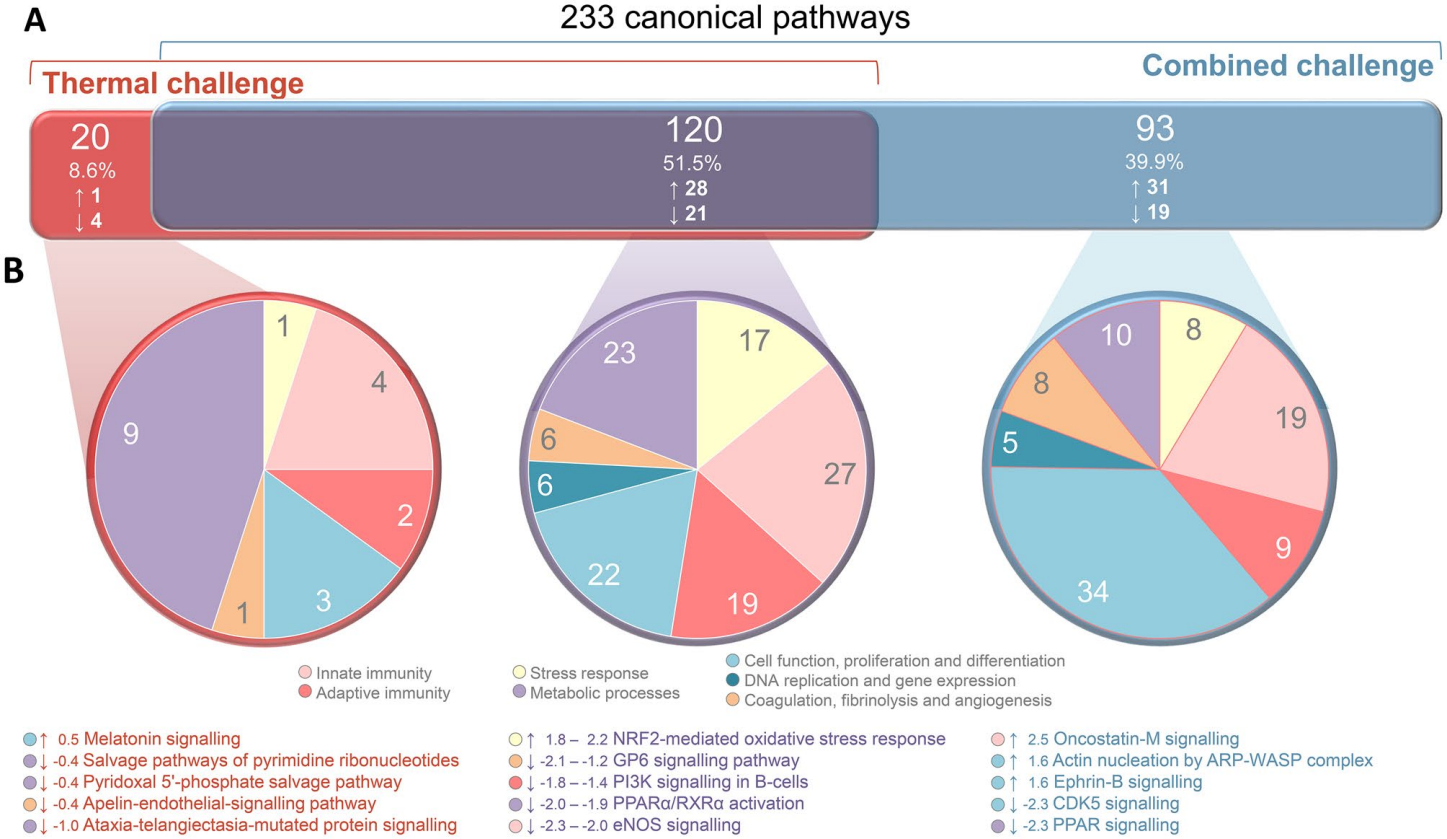
Classification of canonical pathways. (**A**) Venn diagrams illustrating the absolute number, the percentage and the number of activated (↑) and repressed (↓) pathways in the spleen of rainbow trout exposed to high temperature (red), or high temperature combined with crowding (blue) based on the 816 DE genes which are unique for one of the challenges. The pathways were sorted according to the same seven biofunction categories introduced in Fig. 3C. The pie charts depict the categorised pathways (**B1**) exclusively affected by thermal challenge, (**B2**) commonly affected by thermal and combined challenge and (**B3**) exclusively affected by combined challenge. The five most strongly affected pathways are listed with their z-score values below the charts.

The exclusive thermal-challenge DE genes were involved in 140 pathways, while the exclusive combined-challenge DE genes were involved in 213 pathways (Fig. 4A). Both pathway sets overlapped by 52% with 120 shared pathways, majorly the *unfolded protein response, the NRF2-mediated oxidative stress response or eNOS signalling* (Fig. 4B, central pie chart). In the thermal-challenge group, only 20 pathways were uniquely modulated, including the activated *melatonin-signalling* pathway and four downregulated pathways (Fig. 4B, left pie chart). Remarkably, almost half of these temperature-affected pathways were related to metabolism (*n* = 9). In the combined-challenge group, 93 pathways were uniquely modulated, a third of which were predicted to be upregulated (*n* = 31) and a fifth of which was predicted to be downregulated (*n* = 19). Most of these combined-challenge-affected pathways were related to cellular functions (*n* = 34) and innate immunity (*n* = 19). Compared to the thermal-challenge dataset, the proportion of metabolic pathways was significantly decreased (from 45 to 11%) in the combined-challenge dataset, while the proportion of cellular functions increased (from 15 to 37%). Moreover, DNA-replication and gene-expression pathways (5%, *n* = 5) were affected by the combined challenge, but not by high temperature alone.

### Impact of combined challenge on the growth of bacteria in trout serum

**Figure 5:**
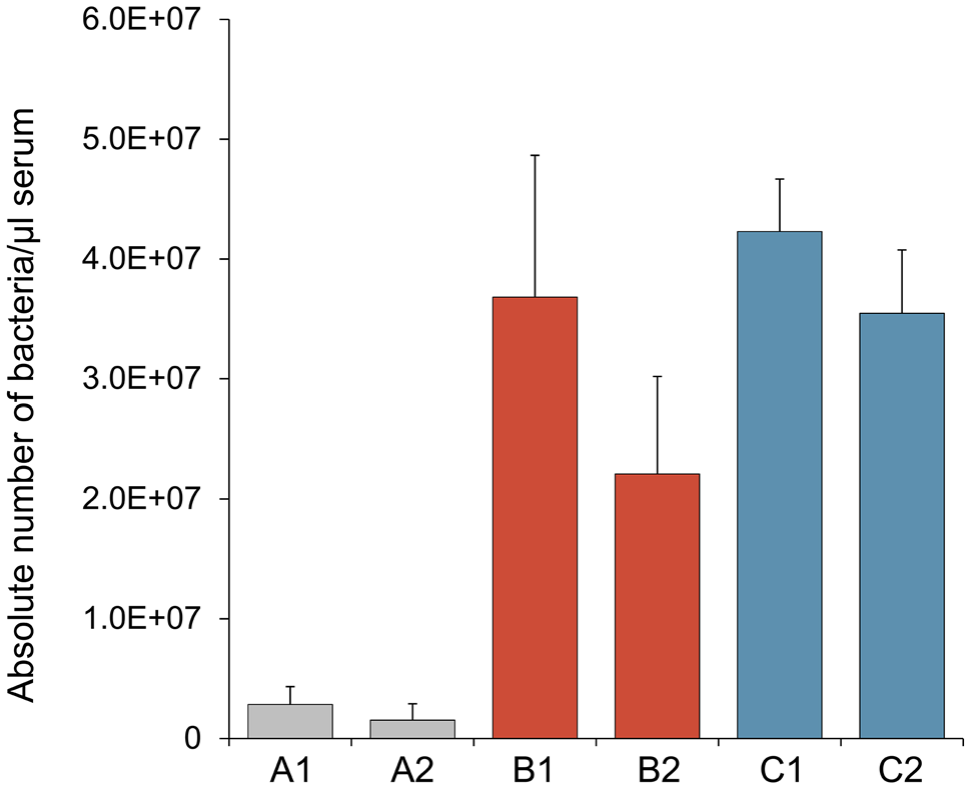
Quantification of the *A. salmonicida* titre in serum isolated from rainbow trout from the experimental tanks as listed along the abscissa (*cf*. Figure 1). Graphs represent the mean values of five fish + SD (error bars).

A common test for assessing immune capacity is to record the growth of bacteria in blood plasma^49,50^. In the plasma of rainbow trout exposed to high water temperature (group B) or high temperature combined with crowding (group C), we counted 7.8 to 27.5 times more *A. salmonicida* after 24-h incubation compared to the bacterial growth in the plasma of reference trout (group A) (Fig. 5).

### Impact of combined challenge on the composition of peripheral blood leukocytes

**Figure 6:**
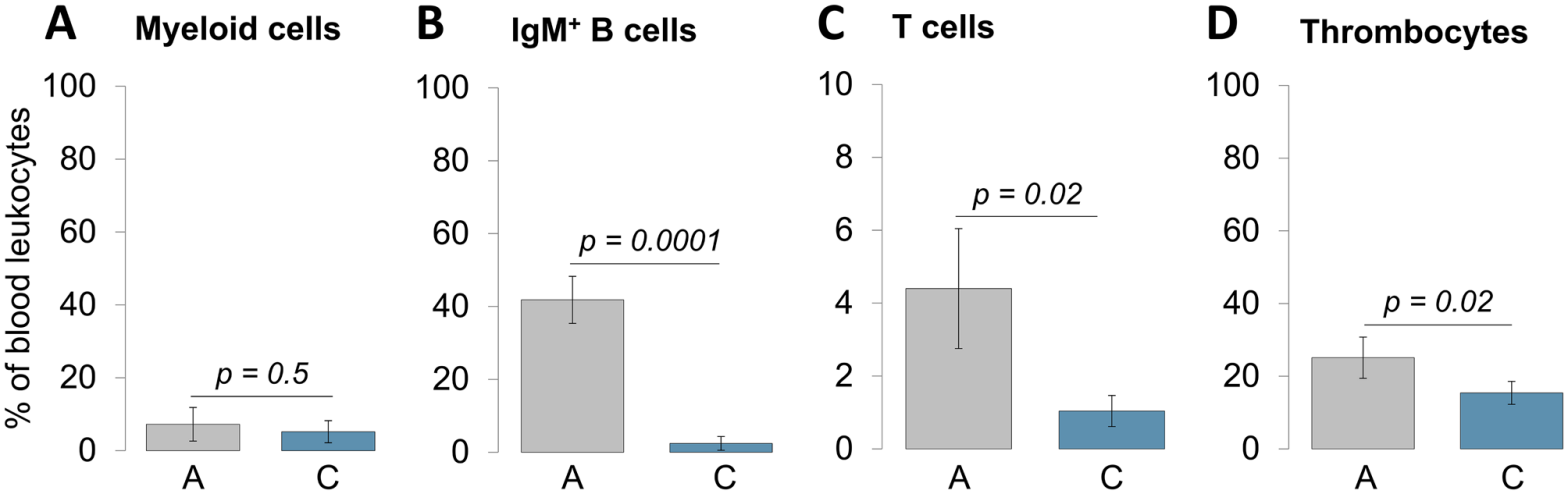
Quantification of the proportion of (**A**) myeloid cells, (**B**) IgM-positive B cells, (**C**) T cells and (**D**) thrombocytes relative to the total amount of blood leukocytes. The cell-sorting procedures were performed with leukocytes from non-challenged rainbow trout (grey bars) and rainbow trout exposed to the combined challenge (blue bars). Graphs represent the mean values of 10 fish (five fish per tank) ± SD (error bars). *P* values are indicated.

Since leukocyte profiles are considered informative stress indicators^51^, we analysed the cell composition of peripheral blood leukocytes in the combined-challenge group (group C) relative to the reference fish (group A). To evaluate the proportion of thrombocytes, IgM-positive B cells, T cells and myeloid cells, we used a previously established flow-cytometry protocol^52^. While the proportion of myeloid cells remained at a similar level (Fig. 6A), the proportion of IgM-positive B cells decreased from 41.8% in the control group to only 2.4% in the combined- challenge group (Fig. 6B). Similarly, the proportion of T cells dropped from 4.4% (group A) to 1.0% (group C) (Fig. 6C), simultaneously with the proportion of thrombocytes decreasing from 25.1% (group A) to 15.4% (group C) (Fig. 6D).

### Expression profiling of immune-relevant genes in stimulated head-kidney cells from challenged versus non-challenged trout

Finally, we determined the impact of previous exposure to critical temperature and crowding on immune responses in vitro. To this end, we isolated head-kidney cells from trout of groups A (reference) and C (combined challenge), stimulated these leucocyte-enriched cultures with *A. salmonicida* and profiled the expression of the inflammation-related genes *TNF*, *IL1B, CXCL8, IL6, TGFB1* and *MHCIIB*.

**Figure 7:**
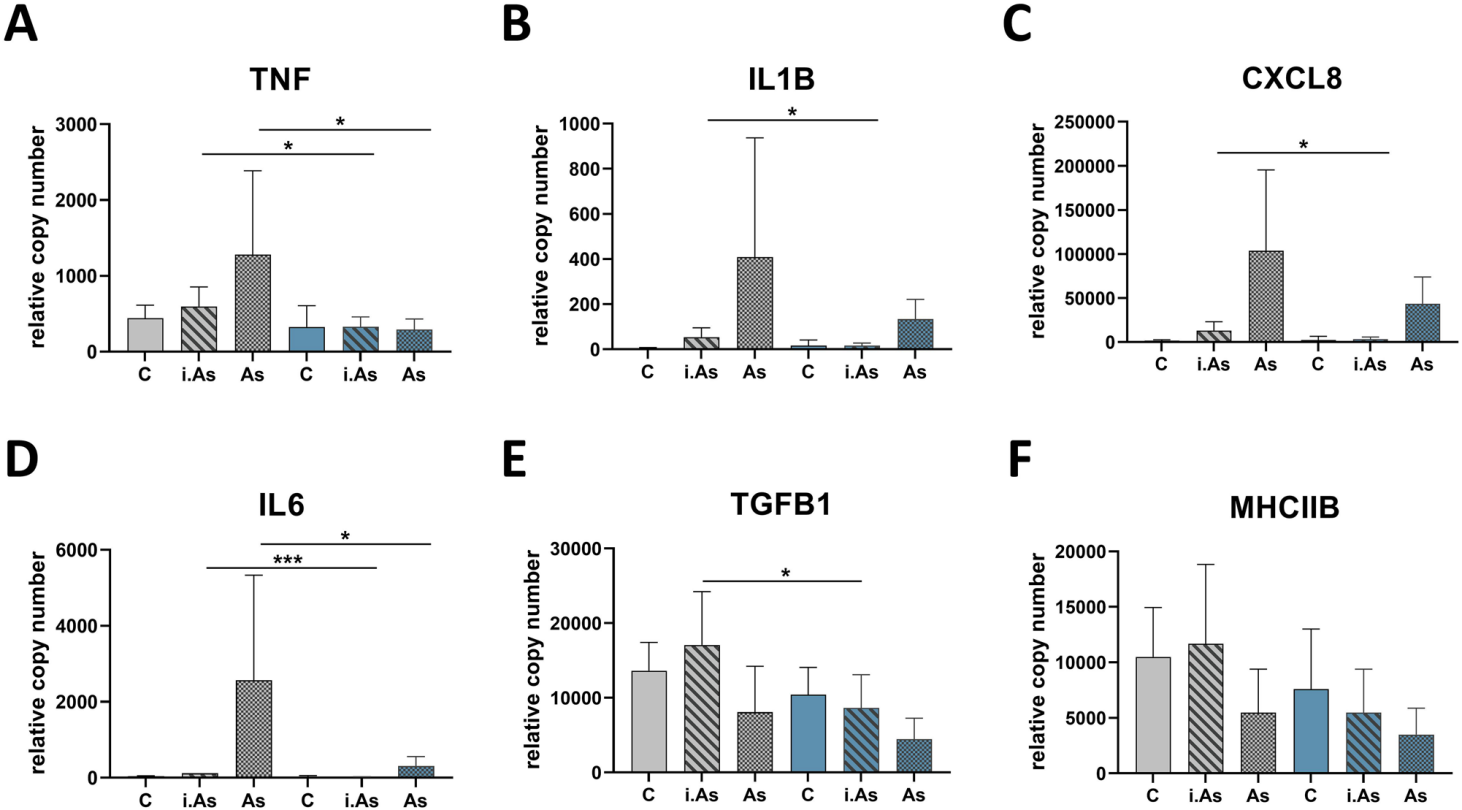
Expression profiling of **(A)** *TNF*, **(B)** *IL1B*, **(C)** *CXCL8*, **(D)** *IL6*, **(E)** *TGFB1*, and **(F)** *MHCIIB* in stimulated head-kidney cells isolated from five non-challenged fish (grey bars) or fish exposed to combined challenge (blue bars). Bars represent the mean copy number + SD (*n* = 8; four fish per tank) normalised to the reference gene *EEF1A1*. Cells from both challenge groups remained untreated (non-textured bars) or they were stimulated with inactivated (i.*As*; hatched bars) or viable *A. salmonicida* (*As*; spotted bars). Significant differences in expression relative to the transcript level in the control group are indicated by asterisks (*, *p* < .05; ***, *p* < .001).

In the head-kidney cells of control group A, the expression of the immune marker genes *TNF*, *IL1B*, *CXCL8* and *IL6* (Fig. 7A–D) increased significantly in response to live *A. salmonicida* while the expression of *TGFB1* and *MCHCIIB* slightly decreased (Fig. 7E,F). A transcriptional response to the stimulation with inactivated *A. salmonicida* was also detectable but less pronounced. The stimulation of head-kidney cells from group C with inactivated *A. salmonicida* did not modulate the transcript levels of the investigated cytokines (Fig. 7, blue bars). In contrast, the stimulation with *A. salmonicida* increased the expression of *IL1B*, *CXCL8* and *IL6*, although to a lower extent as observed for control group A.

## Discussion

### The persistent challenge of rainbow trout in the present study is reflected by distinct expression profiles, but not by altered condition indices and blood parameter levels

The rapid and reliable detection of an informative set of (salmonid-specific) bioindicators allows conclusions on the nature and severity of a multifactorial stress burden and thus significantly improve animal-welfare concepts and the health of farmed fish in aquaculture facilities. The objective of the present study was to discover novel animal-based biomarkers and evaluate well-known existing ones based on transcriptome profiling and to derive inferences about the onset of suggestive pathways. The combination of different techniques, which allow quantifying multiple parameters with relevance to distinct stress responses, is established as a promising approach to assess the individual welfare status^53^.

The present study was based on the recording of primary (cortisol level), secondary (glucose level, haematocrit, leukocyte count, gene expression) and tertiary (condition factor, spleen-somatic index, cellular immune competence) stress indicators. Notwithstanding our expectations, the levels of the analysed non-transcript-based parameters were non-significantly different between the reference (A) and challenge groups (B, C), which might indicate similar physiological states of the animals investigated. The body-score values, in particular, were in the physiological range^54–56^. In stark contrast, the microarray analysis and subsequent experiments with primary cell cultures from control and challenged fish indicate extensive physiological reactions following the experimental challenge. This apparent contradiction is likely to have arisen from the sampling time point chosen. The duration of our experiment ranged between “short-term” and “long-term” and might correspond to a state well after the acute response to the disturbed homeostasis but presumably before the desensitisation to (a) stressor(s) associated with the adaptation processes. The plasma cortisol level, for instance, is undoubtedly an informative indicator of acute stress but in rare cases for chronic stress^57^. The same applies to the glucose concentration^58^. The blood glucose concentrations of all animals examined here were slightly above normal^59^ and most likely reflect our ad-libitum feeding regime. Whereas glucose levels should therefore not be overestimated as an indicator of stress in the present study, the measured cortisol concentrations indicate a general activation of the hypothalamus–pituitary–interrenal axis, commonly known as the stress axis. Although strikingly different cortisol levels have been reported in *Oncorhynchus* spp. under pre-stress conditions (< 2 – > 500 ng/ml)^5^, a concentration below 10 ng/ml is generally considered physiological^60^, and a concentration above 40 ng/ml indicates stress^58,60^. In the present study, the elevated cortisol concentrations likely do not mirror the exposure to adverse temperature and stocking density but rather the acute stress of the sampling procedure including netting, transfer and anaesthesia^61–63^, accompanied by significant inter-individual variabilities. However, the transcription of genes involved in acute stress responses takes a significantly longer period of time^64^ than is required to catch and anaesthetise fish. For this reason, the quantification of transcript levels is supposed to be the more suitable technique in the present study to assess the long-term responses to adverse environmental conditions. If another, uncontrolled factor elevated the cortisol concentration in control and challenged trout, we cannot exclude that this unknown factor also influenced the expression profiles of the three treatment groups and weakened the expression differences.

Regarding the critical temperature of 27 °C and the no less-critical stocking density of 100 kg/m^3^, it would be expected that several functional pathways are induced, which are related to the heat shock, increased energy demand and altered immune defence. The questions remain (1) whether the clear transcriptional responses observed on day eight after the onset of the heat challenge and the heat-crowding challenge, respectively, attenuate or augment over a further extended challenge period and (2) to what extent the presently recorded transcript levels are representative for the long-term exposition to these challenge factors. We can only speculate that the persistent challenge with such an extreme water temperature and the inconvenient stocking conditions will increasingly display various tertiary indicators on the whole-body performance, which would indicate serious detrimental effects and elevated mortality of the rainbow trout.

### Critical temperature and crowding interact synergistically

Our transcriptomic analyses of the rainbow trout spleen revealed that the combination of high temperature (27 °C) and high stocking density (100 kg/m^3^) results in an increased number of DE genes by 162% compared with high temperature (27 °C) alone. Of these, the proportion of upregulated genes was 1.3- to 1.7-fold greater than the proportion of downregulated genes. It is hardly surprising that almost half of the overall regulated genes were present in both sets, as the two challenge groups have been exposed to high temperatures, which are known as major stressors for poikilothermic fish. Nevertheless, we identified two sets of uniquely regulated genes that characterise the response to either high temperature alone or high temperature combined with crowding. Remarkably, the number of DE genes unique to the combined challenge is more than four times higher than the number of DE genes unique to the thermal challenge. This suggests a synergistic interaction of the two challenge factors, especially since our previous study on stocking density in rainbow trout^36^ under similar experimental conditions provided only between one (kidney) and a maximum of 249 (liver) regulated genes, depending on the analysed tissue. The synergistic effect of high temperature and overstocking on the rainbow trout’s physiology is well in line with a meta-analysis by Crain and colleagues, who concluded from 171 studies that multiple stressors generally interact synergistically in marine systems^3^. In this context, the question arises as to which threshold value the stressors must exceed to produce synergistic effects and—in the present case—if the combination of a lower water temperature and/or lower stocking density would have generated additive instead of synergistic effects.

### The signature of thermal stress is conserved across teleost fishes

A considerable number of studies have already profiled the transcriptional changes of different organs of salmonids leaving their thermopreferendum^23,24,27,28,30,65–69^. We^69^ and others^24^ have recently reviewed our own and external transcriptome studies to identify biomarkers of salmonid fish, which indicate a stress response to inconvenient temperatures. Both studies agree that mainly *SERPINH1/HSP47*, *HSPA1A/HSP70* and *HSP90AA1/HSP90* have enormous potential as “robust thermally-responsive biomarkers” following many other reports on these three heat-shock proteins^23,25,27–30,65–68,70–74^. In contrast to the regulation of HSPs, *CIRBP* is strongly downregulated in salmonid fish upon heat stress^23,24,27,69^, while members of the FK506-binding protein family show distinct expression profiles with *FKBP5* being upregulated and *FKBP10* being downregulated^24,69^. Five of these six biomarker genes (*SERPINH1*, *HSPA1A*, *HSP90AA1*, *CIRBP*, *FKBP10*) were significantly regulated by both the thermal challenge and the combined challenge, while *FKBP5* emerged exclusively after combined challenge.

Based on the published DE gene sets responding to heat stress in salmonids, numerous indicative functional pathways were identified. Interestingly, we found that the *NRF2-mediated oxidative stress response* and the *unfolded protein response* were the dominant pathways to responding to critical temperature. The combined challenge in the present study and both pathways were also highly affected after gradual and acute heat stress in different tissues of maraena whitefish^69^. Only 20 pathways were uniquely regulated in response to a high temperature, i.e., they are not present in the list of genes that were differentially regulated in response to the combined challenge. Of these, only *melatonin signalling* was predicted to be positively regulated. This pathway has been described to improve the oxidative status of fish cells, for instance after the activation of reactive oxygen species^75^.

### The signature of crowding is subtly contained in the identified gene lists and pathways upregulated upon combined challenge

Far less is known about those genes characteristically regulated by overstocking in (salmonid) fish. Remarkably, comparative analyses showed that rainbow trout, which have been domesticated for decades in intensive aquaculture, respond considerably less sensitively to crowding than the salmonid maraena whitefish (*Coregonus maraena*), which was only recently introduced in aquaculture^9,36^. *Hypoxia-inducible factor 1-alpha (HIF1α) signalling* was the only pathway found to be activated in the liver of rainbow trout exposed to a high stocking density of 120 kg/m^3,36^. After the combined challenge in the current study, we also identified this pathway regulated in the spleen. Two other pathways, the *protein ubiquitination pathway* and *PI3K/AKT signalling*, were repressed in the liver of rainbow trout exposed to a high stocking density^36^. It is worth noting that in the present study, we found these pathways with an undefined or slightly positive z-score (which corresponds to a slight activation) in trout challenged with a high temperature and crowding. In the combined challenge, 93 pathways were uniquely regulated. Of these, significantly more pathways were activated than suppressed, and a third of these genes are associated with innate immunity (*oncostatin-M signalling*, *NF-κB signalling*, *STAT3 pathway, toll-like receptor signalling*) or adaptive immunity (*FcγRIIB signalling in B cells*, *NUR77 signalling in T cells*).

### Critical temperature combined with crowding impacts a number of immunological pathways and translates to substantial changes in the immune capacity of rainbow trout

Various stressors have been reported to threaten the health of farmed fish in aquaculture. For this reason, it is particularly interesting to investigate the crosstalk between the immune system and stress responses in farmed fish. In our previous study on maraena whitefish, we demonstrated that a high stocking density of 100 kg/m^3^ increased a considerable number of immune-relevant genes^9^. A quarter of all upregulated genes in the liver and a fifth of all upregulated genes in the kidney of challenged maraena whitefish were related to immunological processes including *complement cascade, acute-phase response signalling, chemokine signalling, B-cell-receptor signalling* and *leukocyte-extravasation signalling.* This set of immune pathways is intriguingly similar to the set of induced immune pathways in the liver of large yellow croaker (*Larimichthys crocea*) exposed high stocking-density conditions^76^. The respective pathways belong to the innate immune system (*complement and coagulation cascades, toll-like receptor signalling* and *chemokine signalling*) and the adaptive immunity (*T-cell, B-cell receptor signalling* and *leukocyte-migration pathway*).

In our current study, we also observed notable changes in the expression of genes involved in various innate and adaptive immune pathways. However, the transcriptional changes caused by the gradual increase of the water temperature mainly activated innate-immune mechanisms such as *CXCL8 signalling* and *production of nitric oxide and reactive oxygen species*, apparently to bring the organism into a “watch out” position in anticipation of an increased pathogen pressure. Notwithstanding, we found a decreased bactericidal activity in the plasma of challenged trout compared with non-challenged trout. The complement system is an important immune complex tasked with the elimination of bacteria^77,78^, and we can only assume that several transcripts encoding complement components were downregulated in the liver (the main site of complement synthesis but not considered in this study).

The analysis of the splenic transcriptome clearly revealed that the exposure to combined challenge modulates both arms of the immunity. High water temperature combined with crowding induced several pathways of the innate immunity (*CXCL8 signalling*, *production of nitric oxide and reactive oxygen species* and *micropinocytosis signalling*), while several pathways of the adaptive immunity were downregulated. Of particular importance is not only the decreased expression of genes involved in the *B-cell-receptor signalling,* but also the negative regulation of the *IL7-signalling pathway*, which contributes to early T-cell development and to mature T-cell maintenance. This was confirmed by the altered composition of peripheral blood leukocytes, chiefly by a reduction of B cells, T cells and thrombocytes in fish exposed to combined challenge compared to non-challenged fish. This is well in line with a large number of studies that identified a common pattern of lymphopenia and neutrophilia in the circulation of various fish species exposed to different types of stress^9,79,80^. Overall, the number of phenotypically identifiable cells from challenged rainbow trout was reduced from about 80% to 25% but unfortunately, the available tools did not allow additional information on the nature of the remaining 75% peripheral blood leukocytes from challenged fish to be obtained.

Previously, we had shown with maraena whitefish that adverse environmental conditions do not only influence the transcriptomic programmes in lymphoid organs and the composition of circulating leukocytes but also negatively impact the organism’s ability to react towards pathogens^9^. Using this established protocol, we demonstrate here that the primary culture of head-kidney cells from rainbow trout exposed to the combined challenge showed a dramatically reduced ability to induce the expression of *IL1B*, *CXCL8*, *TNF* and *IL6* upon stimulation with both viable and inactivated *A. salmonicida*. The extent of this downregulation becomes even more evident in comparison to the less pronounced changes induced by crowding alone in maraena whitefish. Taken together, our immunologic analyses confirm the conclusions drawn from our transcriptomic data, which suggest that critical temperature and crowding together have a synergistic effect, especially on the immune capacity of challenged fish. Although rainbow trout were not exposed to pathogens in the present experiment, we assume that an increase in opportunistic pathogens would lead to severe infections and increased death rates of acutely stressed trout. This hypothesis should be verified in future experiments.

## Conclusions

It has been established that temperature and crowding stress have different molecular signatures, which reflect different mechanisms of adaptation. So far, very few data sets have been interpreted concerning the question of how different adaptation mechanisms to simultaneous stressors influence each other. The present study demonstrates that critical temperature combined with crowding activates synergistically a cluster of compensatory responses including cell-signalling, immune and metabolic pathways. Besides, we document that a high temperature combined with overstocking impair the bactericidal and inflammatory activity, significantly alter the blood-cell composition and exert thus a profound impact on the fish’s immune system. Stress does not only compromise the health and general well-being of fish but also reduces the profits of a fish farm.

Our study suggests, once again, to carefully select the parameters for monitoring the response of the fish to one or multiple potential stressors, which are effective over longer periods. High-throughput methods such as RNA-sequencing or microarray analysis allow stress-specific signatures of certain tissues and even cells to be recorded. Although the transcriptome analyses might add valuable information into the physiological events involved in restoring homeostasis, these methods are usually cost-intensive. This study suggests some transcriptomics-based rainbow trout-specific biomarkers that may be relevant for indicating compromised well-being, although they need to be evaluated in an aquaculture practice. Robust parameters resulting from this evaluation can be quantified in a range of specimens through simple and cost-effective methods such as quantitative real-time PCR and extend the available spectrum of welfare biomarkers.

## Materials and methods

### Experimental design and sampling

Rainbow trout at the age of ~ 10 months were randomly distributed over six experimental 309-l tanks integrated into a RAS. The experimental procedures are illustrated in Fig. 1. The stocking density in four of the six experimental tanks (A1, A2, B1, B2) has been set to 30 kg/m^3^ (corresponding to 38 or 39 trout each weighing 9.5 kg), while the stocking density in the other two tanks (C1, C2) was 100 kg/m^3^ (corresponding to 96 or 119 trout weighing 35 kg). Two of the tanks with 30 kg of trout per cubic metre (A1, A2) served as control tanks and were kept at a constant temperature of 16 °C for the duration of the experiment (Fig. 1, reference). The water temperature in the other tanks (B1, B2, C1, C2) was elevated by 2 °C every day for 5 days, gradually going from 16 to 26 °C. On day six, the temperature was increased by 1 °C, and on day seven, the tanks were kept at 27 °C for 24 h. The water temperature and other relevant parameters including NH_4_^+^, NO_2_^−^, NO_3_^−^ (in each case far below critical values), pH (between 7.3 and 8.5) and dissolved O_2_ were constantly recorded. Trout were fed ad libitum commercial dry pellets by automatic feeders. Throughout the experiment, no technical problems were registered. Further husbandry conditions are detailed in reference^36^. Eight days after the start of the experiment, seven rainbow trout per tank (A1, A2, B1, B2, C1, C2) were randomly sampled using hand nets. The fish were quickly transferred to a bucket of water and sedated using an overdose of phenoxyethanol. The handling of fish before blood and tissue sampling was conducted in compliance with the terms of directive 2010/63/EU on the protection of animals used for scientific purposes. The experimental protocol was approved by the Ministry of Education, Youth and Sports, Prague, Czech Republic (approval ID: MSMT-18301/2018–2).

The rainbow trout had reached an average length of 26.2 ± 1.3 cm (mean ± standard deviation, STD) and an average weight of 230.2 ± 35.9 g at the end of the experiment. Blood was taken from the caudal vein of every trout killed using 1-ml plastic syringes filled with 500 µl 0.5 M ethylenediaminetetraacetic acid (EDTA, pH 8.0) solution as anticoagulant. The spleens of the fish were isolated, snap-frozen in liquid nitrogen and stored at − 80 °C until nucleic acid extraction. Head-kidney tissue was homogenised, passed through a 100-µm strainer and washed with cold Dulbecco’s Modified Eagle’s Medium (DMEM, Thermo Fisher Scientific). The resulting cell suspension was loaded on a Percoll gradient (density 1.08 g/ml) and centrifuged for 20 min at 800×*g*. Following centrifugation, cells at the interphase were collected, washed twice in DMEM and resuspended to a final concentration of 5 × 10^6^ cells/ml.

### Blood-parameter analyses

Caudal blood samples from 42 fish were thoroughly mixed with ethylenediaminetetraacetic acid (EDTA) and processed in the haematological counter ABX Pentra 60 (HoribaABX Diagnostics) within 3 h after collection to determine the individual haematocrit and haemoglobin levels.

The residual blood samples were centrifuged (4 °C, 1,700×g), and the supernatant was kept on ice until analysis of blood plasma parameters. Cortisol concentrations were determined using a Cortisol ELISA assay (DRG Instruments GmbH) and plasma-glucose concentrations were measured using a colorimetric assay (Glucose Assay Kit II; BioVision). Both analyses were performed according to the manufacturer’s instructions using a Beckman Coulter DTX 800/880 Series Multimode Detector (Beckman Coulter), which measured the absorbance at 450 nm.

### Bacteria growth serum

For the bacterial growth assay, the individual blood samples of trout were incubated in reaction tubes for up to 1 h. After centrifugation (1,700×*g*, 10 min), 100 µl of serum from each fish was inoculated with 2 × 10^5^ bacteria *Aeromonas salmonicida* ssp*. salmonicida* wild-type strain JF2267. Sera with bacteria were incubated 24 h at 18 °C. After incubation, the bacteria culture from each well was transferred to 1.5-ml tubes and centrifuged at 7,500 rpm for 10 min. The supernatant was discarded, and the total DNA was isolated from a pellet using a QIAamp DNA Blood Mini Kit (Qiagen) according to the manufacturer’s protocol.

### Flow cytometry

The composition of peripheral blood leukocytes was analysed using the protocol established previously^52^. Briefly, 2 µl of heparinised blood was stained with the set of monoclonal antibodies (MAb) recognising thrombocytes (MAb 42–APC-Cy7)^81^, myeloid cells (MAb 21–RPE)^52^, IgM-heavy chain (MAb 1.14–PerCp) and T cells (MAb D30–FITC). The samples were measured on FACS Canto II and analysed by DIVA software (BD Biosciences).

### In vitro stimulation experiment

To investigate the effect of the combined challenge on the immune capacity of rainbow trout, a stimulation experiment with *A. salmonicida* (JF 2267) was performed. The bacteria were kept viable or inactivated in 1.5% paraformaldehyde (PFA) for 1 h and diluted to a final concentration of 5 × 10^7^ cells/ml in sterile phosphate-buffered saline (PBS). Isolated head-kidney cells of eight fish from the reference group A and the combined-challenge group C each were stimulated with 100 µl PBS, 1 × 10^6^ viable or PFA-inactivated bacterium. After 12 h incubation (CO_2_, 15 °C), stimulated samples were collected and stored in 700 μl RLT buffer until RNA preparation.

### RNA preparation

TRIzol reagent (Invitrogen) in combination with the RNeasy Mini Kit (Qiagen) and the RNase-free DNase Set (Qiagen) was used to extract high-quality RNA from the above-listed tissues. The concentration and purity of the RNA was determined using the NanoDrop 1000 Spectrophotometer (NanoDrop Technologies/Thermo Fisher Scientific); RNA integrity was evaluated using the Agilent 2100 Bioanalyzer (Agilent Technologies) revealing values for RNA integrity ranging from 8.9 to 10.

### Array hybridisation

For microarray-based gene-expression analysis, we pooled equal shares of seven individual RNA samples isolated from the spleen in six separate specimens according to treatment and tank (A1, B1, C1; A2, B2, C2). These six RNA pools were converted to Cy3-labelled cRNA and hybridised with 8 × 60 K Agilent-049158 Salmon Oligo Microarrays (Agilent Technologies; GEO platform: GPL21057) following the Agilent 60-mer oligo microarray processing protocol, as described in our previous paper^36^. The fluorescence signals of the hybridised Agilent microarrays were scanned with a G2505C Microarray Scanner System (Agilent Technologies) at a resolution of 3 µm.

For all hybridisations, two technical replicates were included representing exactly the same samples but applied to independent arrays. The reliability of the microarray-predicted data has previously been proven in various quantitative PCR studies of our group^9,23,69,82,83^.

### Quantitative real-time PCR analysis

Quantitative real-time PCR was performed using the LightCycler96 Real-Time PCR System (Roche) and SensiFAST SYBR No-ROX One-Step Kit (Bioline). RNA samples from the in vitro*-*stimulation experiment were reverse-transcribed using the Super Script II kit (Thermo Fisher Scientific) and a cDNA equivalent of 75 ng total RNA was analysed for copy-number analysis. Gene-specific primers for the immune-related genes *CXCL8*, *IL1B*, *TGFB1*, *TNF*^84^, *IL6*^85^, and *MHCIIB* (sense, 5′-AGTACACACCCAAGTCTGGAGA-3′; anti-sense, 5′-AGTCCTGCTAATGCTAAGATGGT-3′) were used.

The number of bacteria was quantified on the CFX96 Touch Detection System (Bio-Rad) using SensiFAST SYBR one-step kit (Bioline) according to the protocol. As target gene, DNA gyrase (subunit B) was used (NCBI gene ID: 4994747; sense, 5′-TCATCATGACTGTGCTGCAC-3′; antisense, 5′-ATGGTCAGCAACAGCTTGT-3′)^86^.

Cycling conditions were as follows: 10 min pre-incubation; 15 s denaturation at 95 °C, 10 s annealing decreasing from 60 °C, 20 s elongation at 72 °C, and 5 s quantification at 75 °C. To assess the quality of individual PCR products, a melting-curve analysis was performed.

### Data analyses

Statistical analyses of body-score indices (condition factor, spleen-somatic index, haematocrit) and plasma parameters were performed in GraphPad Prism 8.4.2 (GraphPad Software, Inc., San Diego, CA) with a significance level of 0.05. Because all data were normally distributed (Kolmogorov–Smirnov, *p* > 0.05), parametric *t* tests were used.

The Agilent Feature Extraction Software (FES) 10.7.3.1 was used to read and process the microarray-image files with standard settings. The FES corrected the background using a two-sided Student *t* test. The features that passed this quality control were further analysed with the limma package of the R-Version 3.1.1/Bioconductor-Suite^87^). Our customised scripts are available on request. Following quantile normalisation, pairwise comparisons between the treatments (reference vs. thermal challenge or combined thermal/crowding challenge) were employed to compare transcript abundances under the different treatment conditions. To control the false discovery rate, *p* values were adjusted^88^. Only genes with a corrected *p* value (*q*-value) of < 0.05, and an absolute fold change of > 2.0 were considered DE genes and included in further data processing.

Comparisons of gene expression across the different treatment conditions were performed using Venn Diagrams^89^. DE genes were re-annotated using the basic local alignment search tool (BLAST). We considered only transcripts with unique BLAST results (coverage and sequence identity of > 85% and E-value < 1 × 10^−4^). Only 2.5% of all DE genes could not be annotated. A functional analysis was performed using the Ingenuity programme (Ingenuity Pathway Analyses/Qiagen) to evaluate global functional networks and canonical pathways of interacting genes and other functional groups.

Benjamini–Hochberg multiple testing was performed and *p* values > 0.05 were considered as a cut-off score. The output lists were carefully reviewed, and all those pathways and functions were deleted which were either associated with a mammalian disease or occurred in a tissue other than analysed. All relevant pathways are indicated in the following sections by italic face. Additionally, we assigned the canonical pathways to the biofunctions “stress response”, “innate immunity”, “adaptive immunity”, “cell function, proliferation and differentiation”, “DNA replication and gene expression”, “coagulation, fibrinolysis and angiogenesis” and “metabolic processes”. If certain pathways were assigned to more than one biofunction, we still only assigned them to one main category. The z-scoring system was used to evaluate whether a particular pathway was activated (*z* > 1) or repressed (*z* < 1).

The number of transcripts determined via quantitative PCR was calculated based on transcript-specific standard curves. To this end, the C_q_ values of PCR products, having been amplified by the aforementioned primer pairs, were determined in serial tenfold dilutions starting from 1 × 10^2^ to 1 × 10^8^ copies. Based on these C_q_ values, a calibration line was created, which allowed for the calculation of the copy numbers according to the formula *copy number* = *10*^*(Cq value* − *intercept)/(slope)*^. The obtained values were normalised to the reference gene encoding the translation elongation factor EEF1A1^90^.

In an analogous manner, the number of *A. salmonicida* in the serum of rainbow trout was determined. The number of cells per ml was calculated based on a calibration line. This has been generated based on the Cq values of the *gyrB* transcripts determined for 1 × 10^4^–1 × 10^9^ colonies.

## Supplementary information

Supplementary information 1.

Supplementary information 2.

## Data Availability

The full complement of microarray data generated and analysed during the current study was deposited in the NCBI GEO, https://www.ncbi.nlm.nih.gov/geo/; accession: GSE129271).
