# Supplementary material for: The synergistic interaction of thermal stress coupled with overstocking strongly modulates the transcriptomic activity and immune capacity of rainbow trout (Oncorhynchus mykiss)
Source: Sci Rep. 2020 Sep 10;10:14913. doi: 10.1038/s41598-020-71852-8 (PMC7483466; doi:10.1038/s41598-020-71852-8)
Supplement: Supplementary file 1 — Supplementary information 1. [file 41598_2020_71852_MOESM1_ESM.docx]

**Supplementary Figure 1:** Temperatures of the brackish coastal water of the southwestern Baltic Sea in Born/Germany in the period from 22/07/2018 to 30/07/2018. Individual values were recorded every 30 minutes.
