# Supplementary material for: The synergistic interaction of thermal stress coupled with overstocking strongly modulates the transcriptomic activity and immune capacity of rainbow trout (Oncorhynchus mykiss)
Source: Sci Rep. 2020 Sep 10;10:14913. doi: 10.1038/s41598-020-71852-8 (PMC7483466; doi:10.1038/s41598-020-71852-8)
Supplement: Supplementary file 2 — Supplementary information 2. [file 41598_2020_71852_MOESM2_ESM.pdf]

Scientific Reports

The synergistic interaction of thermal stress coupled with overstocking strongly modulates the transcriptomic activity and immune capacity of rainbow trout (*Oncorhynchus mykiss*)

Alexander Rebl,[1] Tomáš Korytář,[2] Andreas Borchel, [3] Ralf Bochert,[4] Joanna Ewa Strzelczyk,[5] Tom Goldammer, [1, 6] Marieke Verleih[1§]

[1] Institute of Genome Biology, Leibniz Institute for Farm Animal Biology (FBN), Wilhelm-Stahl-Allee 2, 18196 Dummerstorf, Germany

[2] Institute of Aquaculture and Protection of Waters, Faculty of Fisheries and Protection of Waters, University of South Bohemia, Husova tř. 458/102, 370 05 České Budějovice, Czech Republic

[3] Sea Lice Research Centre (SLRC), Department of Biology, University of Bergen, Thormøhlensgate 55, 5008 Bergen, Norway

[4] Institute of Fisheries, State Research Centre of Agriculture and Fisheries, Mecklenburg-Vorpommern (LFA-MV), Südstraße 8, 18375 Born, Germany

[5] Institute of Immunology, Federal Research Institute for Animal Health, Friedrich-Loeffler-Institute, 17493 Greifswald-Insel Riems, Germany

[6] Faculty of Agriculture and Environmental Sciences, University of Rostock, 18059 Rostock, Germany

§ Correspondence:
Dr. Marieke Verleih
Institute for Genome Biology
Leibniz Institute for Farm Animal Biology (FBN)
Wilhelm-Stahl-Allee 2
18196 Dummerstorf, Germany
E-mail: verleih@fbn-dummerstorf.de
Phone: +49 38208 68721

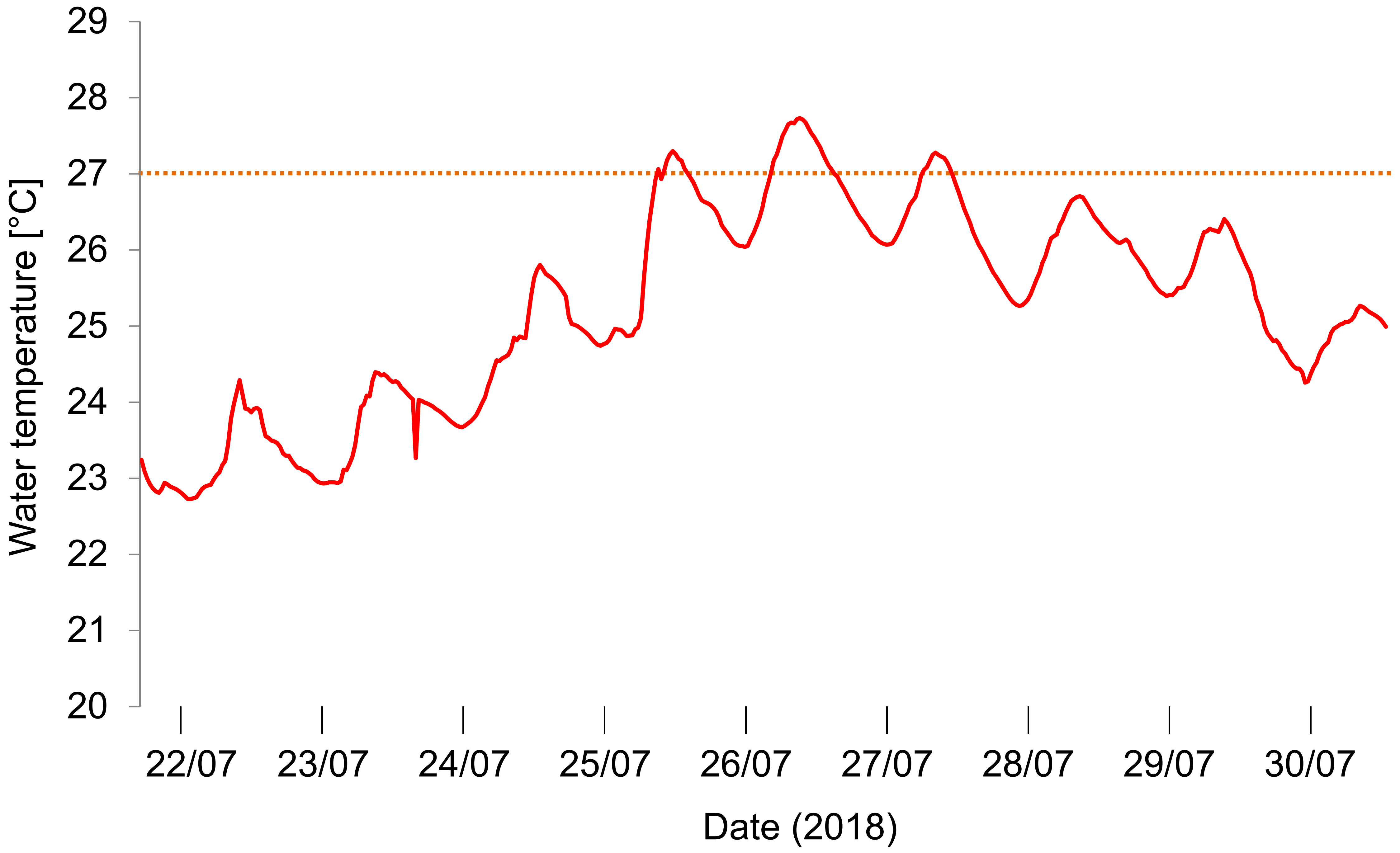
29
28
27
26
25
24
23
22
21
20
Water temperature [°C]
22/07
23/07
24/07
25/07
26/07
27/07
28/07
29/07
30/07
Date (2018)
